# Analysis of Emergency Department-based Intensive Care Units on Coding and Revenue

**DOI:** 10.5811/westjem.41521

**Published:** 2025-09-27

**Authors:** Michael H. Sherman, Vincent L. Kan, Patric Gibbons, Jacob Garrell, Martin A. Reznek

**Affiliations:** * Boston University Chobanian & Avedisian School of Medicine, Department of Emergency Medicine, Boston, Massachusetts; † University of Massachusetts Chan Medical School, Department of Emergency Medicine, Worcester, Massachusetts; ‡ University of Massachusetts, Department of Anesthesiology & Perioperative Medicine, Worcester, Massachusetts

## Abstract

**Introduction:**

Emergency department-based intensive care units (ED-ICU) address the increasing demand for critical care services and represent a transformative approach to the specialty’s management of critically ill patients within emergency medicine. However, data on their financial impact and operational effects remain limited.

**Methods:**

We conducted a retrospective, quasi-experimental study at an urban, academic ED with approximately 90,000 annual visits. In July 2019, a nine-bed ED-ICU model, referred to as “Next Pod,” was implemented. We analyzed Current Procedural Terminology (CPT) coding data and professional revenue (charges billed and payments received) for 35 weeks before and after the intervention (November 2018–March 2020). The intervention involved repurposing a nine-bed ED area and adjusting physician and nursing staffing models. We compared critical and non-critical care CPT coding proportions and professional revenue using the Student *t*-test.

**Results:**

During the study period, there were 38,283 ED visits pre-implementation and 36,424 visits post-implementation. Across the entire ED, critical care coding significantly increased following implementation (CPT 99291: 6.2 – 8.8% [total percentage increase of 41.94%]; 99292: 0.5 – 1.0% [total percentage increase of 100%]). Encounters where 99292 was billed multiple times increased by 128.1% (32 vs 73). Non-critical care coding (99282, 99283) decreased 23% (9.1% vs 7.0%, P< .001) / 29.6% (16.2 vs 11.4, P < .001), respectively. There was a non-statistically significant increase in 99284. Higher acuity codes (99285) increased by 10% (31.7% vs. 34.9%, P < .001). Average ED charges per visit increased by $40 (95% CI $37.2 – $45.5) post-implementation..

**Conclusion:**

The implementation of an ED-ICU was associated with significant increases in critical care and high-acuity coding, as well as enhanced professional revenue. These findings suggest that ED-ICU models can improve both fiscal performance and operational efficiency. Further research is needed to explore the contributions of resource allocation, documentation improvements, and care practices to these outcomes.

## INTRODUCTION

The integration of intensive care units within emergency departments (ED-ICU) represents a transformative approach to the specialty’s management of critically ill patients.^1^,^2^ This innovation is grounded in the need for rapid, specialized care amidst rising patient acuity and volume, alongside prolonged boarding times for critically ill patients in the ED.^3^ This has prompted several operational innovations, including different ED-ICU models within various EDs in the United States.^4^ First described in 2013, there are several different ED-ICU models that seek to address the increasing demand for immediate critical care services in the ED.^1^ These units remain a growing area of ED quality improvement as each was designed to meet its specific institutional needs.^4^,^5^ While ED-ICUs are usually part of an ED, and thus not licensed or staffed as ICUs, they are generally focused on providing prompt acute care and increased resources during the early resuscitation and acute care afterward.

These ED-ICU models have been associated with a reduction in risk-adjusted, 30-day mortality among all ED patients, decreases in hospital and 24-hour mortality, and a reduction in ICU admissions from the ED, especially with a reduction in short-stay ICU admissions.^6^ The various ED-ICU models’ effects on improved healthcare delivery have been demonstrated.^6^–^13^ However, data are limited regarding the financial performance of these units, and concerns regarding their fiscal viability have been cited.^7^,^14^

Following a quality and flow improvement initiative supported by ED and hospital leadership to improve provision of care, our study site implemented a novel, hybrid-model resuscitation care unit as part of the ED, offering an opportunity to explore financial outcomes associated with such an ED operational change.^4^ Our primary objective in this study was to assess the effect of an ED-ICU model and its association with overall changes to ED professional services coding (Current Procedural Terminology [CPT], billing [charges generated], and realized fees for professional services [payments received]). We hypothesized that the integration of an ED-ICU model into the ED to care for high-acuity visits would result in a shift toward increased critical care CPT coding, resulting in increased professional revenue (both charges and payments).

## METHODS

### Study Design and Setting

We conducted a retrospective, quasi-experimental study at an urban, academic medical center in the US with approximately 90,000 annual ED encounters. The institutional review board at the University of Massachusetts Chan Medical School reviewed and approved this study.

### Selection of Participants

On July 15, 2019, a nine-bed unit dedicated to the care of critically ill and other high-acuity patients was integrated into the adult section of the ED. The unit was referred to as the “Next Pod,” short for “North Pod Extension” given its geographic proximity to the “North Pod” of the ED. We examined CPT coding and billing data 35 weeks before and 35 weeks after the implementation of Next Pod (November 11, 2018–March, 15, 2020) of all patients ≥18 years of age who had at least one ED encounter. The study period was chosen because of the unanticipated effect of the COVID-19 pandemic on ED visits, which substantially affected the study site’s ED volume starting in March 2020 and limited the timeframe available for meaningful data collection and comparison that would otherwise have been included.

**Population Health Research Capsule**

What do we already know about this issue?

*ED-ICUs improve clinical outcomes, but their impact on billing practices and revenue generation has not been well characterized*.

What was the research question?

*Does implementing an ED-ICU change coding patterns and increase professional revenue?*

What was the major finding of the study?

*CPT 99291 to bill for critical care rose from 6.2% to 8.8%, 99292 from 0.5% to 1.0% (P < .001); charges/visit increased by $40 (95% CI $37.2 – $45.5, P < .001)*.

How does this improve population health?

*Observed increases in revenue generation may reflect better documentation and efficiency, supporting scalable, sustainable ED-models to improve healthcare delivery*.

### Measurements

Demographic variables collected included basic characteristics including age, sex (as identified by patient), race and ethnicity, and payor. We used the Emergency Severity Index (ESI) to adjust the results for disease severity variation between pre–Next Pod and post–Next Pod implementation groups.^15^ In accordance with best practices for retrospective chart review studies, as outlined by Worster and Bledsoe,^16^ this study adhered to key methodological standards. These included a clearly defined research question, standardized data collection protocols, a well-defined study population, and the use of validated measures such as CPT codes and professional revenue data. Data were sourced from a centralized administrative database to ensure consistency and accuracy.

### Interventions

Following a dedicated multidisciplinary ED quality and flow Kaizen event to identify potential improvement initiatives, the study site implemented a dedicated nine-bed ED-ICU model in July 2019. The Next Pod was created by repurposing a smaller nine-bed area of the ED (~2,653 sq ft), with no physical space or floorplan change to ED layout. Capital improvements were limited, mainly comprised of the addition of a Pyxis MedStation. No specialized supplies were purchased or stocked in the Next Pod.

The Next Pod unit was continuously staffed by one attending emergency physician and one postgraduate year 2 or 3 emergency medicine (EM) resident. This represented an increase in staffing by an additional attending 24 hours per day. Only two ED faculty were board-certified in critical care and EM during this period. The EM-critical care faculty had a higher percentage of Next Pod shifts than non-critical care faculty, although the difference was minimal compared to total coverage. The overall daily resident staffing remained relatively unchanged by adjusting resident staffing models to optimize learning opportunities and in a budget-neutral fashion. This physician team would also respond to cover a dedicated resuscitation area (trauma bays) of the ED to perform the initial evaluation and management of trauma, ST-elevation myocardial infarction (STEMI) and acute stroke activations as well as patients with cardiac arrest in the field.[Fig f1-wjem-26-1192][Fig f2-wjem-26-1192]

The Next Pod team managed high-acuity traumas, STEMIs, and strokes, providing care in the trauma bay until definitive treatment (eg, operating room, interventional radiology, catheterization lab), and then transitioning patients to Next Pod for continued monitoring, or handing off to other ED pods as needed; responsibility for patients remained with the Next Pod team until patients were physically transferred to other ED areas. On resident educational days, the ED-ICU was staffed by the ED attending without a resident for six hours. The unit was staffed with a minimum of three nurses, representing an increased patient-to-nurse ratio at a minimum of 3:1. Generally, patient-to-nurse ratios were ~5:1 in other areas of the ED. No additional training was required for nursing staff to care for patients in Next Pod; however, in general more senior/experienced ED nurses were assigned to work in the unit.

This Next Pod functioned as an integral part of the ED, and patients were triaged to or transferred to and from other parts of the ED depending on acuity and changes in clinical status. No specific conditions, treatments, or diagnoses mandated triage or transfer to Next Pod. In general, Next Pod cared for high-acuity medical and critical polytrauma patients presenting to the study site ED. Many of these patients were acutely ill on a background of chronic illness and often carried significant medical comorbidities typical of a tertiary-care facility. Due to limitations in the dataset abstracted from the billing network, and technical constraints of the electronic health record, we were unable to accurately track patients who were transferred into Next Pod from other areas of the ED. However, this area primarily received patients with Emergency Severity Index (ESI) scores of 1–2, and occasionally 3. Patients were either directly triaged to Next Pod upon arrival based on the severity of illness or were transferred there after being identified as critically ill during their ED course. Thus, the primary intervention was a model that increased nursing ratios and physician coverage, enabling more intensive patient management in a dedicated space that followed standard EM principles, focusing on acute care to optimize treatment for high-acuity patients and maintain ED workflow. No formal coding or billing educational program was implemented for the Next Pod initiative before, during, or after implementation. The department did engage in regular reviews of billing code distributions at staff meetings related to relative value unit (RVU) data; however, this was a longstanding initiative and was consistent and unchanged during and around the study period.[Table t1-wjem-26-1192]

The study site was a Level 1 trauma center, a certified Advanced Comprehensive Stroke Center, and a percutaneous coronary intervention center (PCI). We identified an affiliated community ED in close geographic proximity (1.7 miles away) to serve as a natural control group for comparison variation between pre-Next Pod and post-Next Pod implementation groups concurrently. This community ED was part of the same hospital system as the study site but not a trauma center. The control and study EDs were located on separate but affiliated hospital campuses. Patients evaluated at the control hospital who required acute stroke, PCI, or trauma intervention and management were promptly transferred to the study hospital campus for those services. On average, <5% of patients evaluated at the control campus ED required transfer to the study hospital campus. The control hospital had inpatient ICU services and most subspecialty services. Most ED patients were admitted to their respective hospitals, except those requiring management of acute stroke, acute PCI, or acute trauma services. Patients with acute transplant-related or acute oncologic issues may have been transferred to the study campus, although these cases were also rare. The natural control group ED was staffed by the same academic ED with nearly all the physicians practicing at that site also practicing at the primary study site.[Fig f3-wjem-26-1192]

### Outcomes

The primary outcome of this study was the change in billing of critical care CPT codes (99291, 99292) following the implementation of Next Pod. Secondary outcomes included changes in billing of non-critical care EM CPT codes (99281–99285) and overall professional revenue. We compared average billing (charges generated per ED visit) and payments (collections received) between the pre- and post-Next Pod implementation cohort to evaluate changes in ED professional revenue over time.

Overall, there were minor Centers for Medicare & Medicaid Services reimbursement changes from 2018–2020 during the study period. Total RVUs in 2018 and 2019 remained stable aside from a 0.4% decrease in 99291 and 99292 total RVUs. Between 2019 and 2020, there was an upward trend in 99281–4 billing, but 99285 work RVU remained unchanged. Overall reimbursement per RVU remains complex and is addressed in the Supplement. However, given the timeframe of small changes, this was felt not to substantially affect outcomes. Inflation was not accounted for as there is no established inflation adjustment in the healthcare sector^7^; it is also adjusted for in annual CMS RVU reimbursement changes (Supplement). Per the US Bureau of Labor Statistics, the consumer price index inflation between November 2018–March 2020 was 2.4%. Given this short period and outcomes, we believe inflation did not substantially affect outcomes.[Table t2-wjem-26-1192]

### Analysis

Descriptive statistics were presented as means ± standard deviations for continuous variables, and categorical variables as percentages. We compared proportions of CPT codes and professional fee revenue before and after implementation. We compared the proportion of each evaluation and management (E/M) code between before and after Next Pod implementation using a series of 2x2 chi-square tests. For each code (99281–99285, 99291, 99292), we tested whether its frequency differed significantly between the two groups. Differences in revenue (via charges and payments, total and per encounter) were examined using the Student *t*-test. Two-tailed values of *P* < 0.05 were considered statistically significant. We performed data analysis performed using STATA/MP v17 (StataCorp, College Station, TX).

## RESULTS

**Figure 4:**
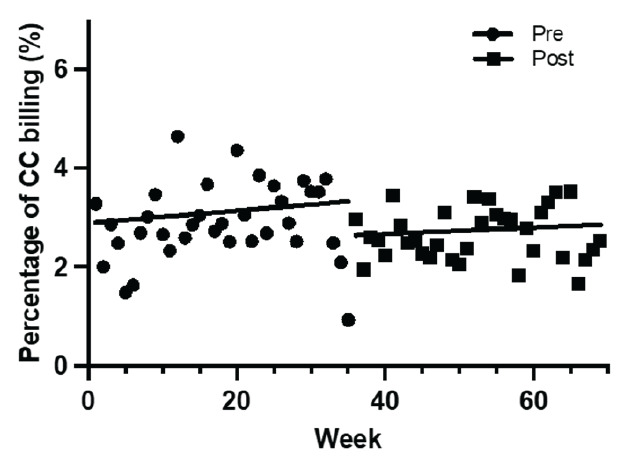
Percentage of emergency department encounters with critical care time billed at natural control site before and after the implementation of Next Pod. Note that Next Pod area is the ED-ICU. *CC*, critical care; *Next Pod*, North Pod Extension; *ED-ICU, e*mergency department-based intensive care units.

There were 38,283 ED visits pre-intervention compared to 36,424 post-intervention during the 70-week total study period. During the post-implementation period, 5,159 patients were triaged initially to the Next Pod, for an average of 19.8 patients per day. Due to limitations in the electronic health record, this did not account for patients transferred to the Next Pod later in their ED course; so, it underestimates the total number of patients cared for in the unit. The mean ESI among all patients before and after implementation (2.84 pre vs. 2.85 post) were similar. The natural control site, an affiliated in-system hospital in close geographic proximity, showed no statistically significant changes in the primary outcome of critical-care CPT billing codes or percentage of total ED encounters with critical-care billing codes pre and post implementation (2.89 vs 3.07) (Figure 4).

In the primary outcome of critical care CPT billing codes at the study site, we found a significant increase in both 99291 and 99292. In the pre-period, 6.2% and 0.5% of all CPT codes billed were 99291 and 99292, respectively. In the post-period, 8.8% and 1.0% of all CPT codes billed were 99291 and 99292, respectively. The implementation of Next Pod resulted in a 2.6% net increase (95% CI [confidence interval] 2.2–2.9%) of 99291 billed (total percentage increase of 41.94%) and a net increase of 0.5% (95% CI 0.4–0.6%) of 99292 billed (total percentage increase of 100%). Encounters where 99292 was billed multiple times increased by 128.1% (32 vs 73).[Fig f5-wjem-26-1192]

In the secondary outcomes of non-critical care CPT codes, we noted a decline in lower complexity codes, and an increase in higher complexity codes. Compared to the pre-period, the total percentage of 99282 decreased by 23% (9.1% vs 7.0%, *P* < .001) and 99283 decreased by 29.6% (16.2 vs 11.4, *P* < .001). At the same time, the percentage of 99285 increased by 10% (31.7% vs. 34.9%, *P* < .001) in the post-period. There was a non-statistically significant increase in 99284. No changes were seen in the proportion of 99281 billed.

In review of charges and payments, we found a statistically significant increase in both post- implementation. We observed a mean increase of charges per encounter of $40.0 (95% CI 37.2 – 45.5, *P* < .001) following the opening of the unit, a 10% increase. We observed a mean increase of payments per encounter of $11.9 (95% CI 10.5 – 13.3, *P* < .001) of all ED encounters following the opening of the unit. There was a $37 net increase per critical care encounter (4% total increase). Although the changes in critical care average charges were not statistically significant (*P* = .08), they did indicate an increased trend post- implementation of the ED-ICU. These trends were noted despite the post-group having a decline in total ED encounters of 4.85% (38,283 vs. 36,424).

## DISCUSSION

In this study we sought to contribute to improved understanding of the fiscal impact of ED-ICU models by examining professional coding and revenue. We found that the integration of our Next Pod unit into the existing ED infrastructure was associated with an increase in the primary outcome of critical care CPT coding (99291, 99292), as well as secondary outcomes of increased proportion of higher complexity, non-critical care coding (higher 99285, lower 99282 and 99283). Associated with this was an increase in charges and payments per encounter for all ED encounters. Charges also increased per encounter for critical care, likely representing an increase in subsequent critical care coding (99292) and by encounters with multiple 99292 codes. This points toward the driver of increased revenue being an increased proportion of high complexity and critical care coding. Our observations in total provide evidence that implementation of these units can result in increased professional revenue generation (both charges and payments).

The significant rise in 99291 coding (initial critical care) may indicate operational enhancements within the ED,—by isolating the most critically ill to be cohorted by acuity while allowing other ED areas to better focus on throughput. This allocation of resources could also allow for more focused care and improved documentation accuracy. This supposition is supported by the trend toward higher acuity EM coding across the ED during the post-implementation period. Following implementation of the unit as part of the local process improvement Plan-Do-Study-Act cycle work, physicians and nurses reported that in the lower acuity areas of the ED, staff perceived that they could better balance their attention across all their patients when not burdened with a critically ill patient requiring prioritization of their attention. Whether the increase in 99291 coding reflected more resources dedicated to critical care or more time and attention for documentation practices across the ED, not just the Next Pod, remains unclear.

The increase in 99292 coding (subsequent critical care) in the post-implementation period represents a revenue stream that appeared to have been underused prior. In addition to single 99292 codes, the increase in ED encounters with multiple 99292 codes likely represented increased longitudinal critical care services provided in the ED. Gunnerson et al demonstrated the effect of an ED-ICU on improved patient-centered outcomes and resource utilization, and is likely reflective of increased bedside care and recognition of the longitudinal treatment of the critically ill.^5^,^6^,^17^ We surmise that the increase in 99292 coding likely reflected additional attending time spent in care of these patients, although it is possible that change in documentation practices may also have occurred with the unit’s implementation (more time to document or heightened awareness of critical care documentation). We suspect both contributed, but considering prior reports, additional time spent by the attendings with these patients was likely a significant component. If so, this increased capture of 99292 billing codes represents a direct acknowledgment of the continuous care of critically ill patients and demonstrates appropriate reimbursement for those services provided in an ED-ICU setting that have been demonstrated to improve outcomes.^6^

Analysis also revealed a shift in non-critical care E/M CPT codes, with a significant decrease in lower complexity code billing not solely explained by the increase in critical care coding. The cause for greater 99285 (higher complexity) coding and billing remains unclear. We have no reason to suspect a change in the patient population or coding and billing practices during the period. The ESI, a surrogate marker for complexity, remained stable during the study period, and there were no departmental interventions related to coding and billing practices.^15^ It is possible this reflected the seasonality of ED presentations, although this remains unclear.^18^,^19^ The rise in higher complexity billing codes after ED-ICU implementation may reflect how exposure to a clinical environment might subtly shape physician behavior.^20^,^21^ This trend may also be influenced by better documentation habits over time vs the seasonal variability in ED visits. As the charge structure remained unchanged, the shift in coding likely reflects a real change in documentation practices rather than financial motivation.

The shift in coding may have reflected improved operations within the ED overall, not just in the care of critically ill patients. This is consistent with prior reports of other acuity-based, split-flow models such as practitioner in triage and/or low acuity “fast-tracks,” which have been associated with improved revenue generation, efficiency, and resource utilization.^22^,^23^ In effect, an ED-ICU-type unit functions as a third-line split flow: triaging or moving patients with high-acuity, resource-intensive conditions to a dedicated area with a different resource allocation. From an operational improvement standpoint, the idea that this would improve effectiveness makes intuitive sense.

Regardless of causality, our findings of increased professional fee generation associated with implementation of the Next Pod unit suggest a positive fiscal impact of ED-ICU care models. The fiscal gains highlighted by our study, particularly the 10% increase in charges per ED encounter ($40.0), signal a notable enhancement in the ED’s revenue stream of $11.9 payments per encounter. Assuming 90,000 encounters/year, this would result in an estimated increase of $1 million per year from professional fee payments alone. Of note, our investigation did not evaluate facility fee coding and billing, but in general these correlate with professional coding and are a multiple greater than the professional fees.^24^–^26^ Furthermore, ED-ICU care models have been shown to increase hospital transfer acceptance rates and provision of critical care and surgical services.^11^,^27^ A comparison study found a decrease in direct costs per ED encounter by 22.1% among critically ill patients, and those authors suggested that providing early, coordinated critical care in an ED-ICU could lead to overall cost reductions by preventing disease progression and complications.^7^ Therefore, we suspect that there was additional revenue and benefits to the hospital associated with the implementation of the unit not measured in our study.

Our investigation focused on charges and payments and did not examine cost association. The literature remains sparse in this regard. The implementation of an ED-ICU model has previously been associated with decreased, risk-adjusted 30-day mortality and reduction in ICU admissions, leading to an overall improvement in resource utilization.^6^ Prior studies by Bassin et al in a foundation-funded ED-ICU demonstrated that their ED-ICU was cost neutral, although their unique funding structure limits generalizability and applicability to other centers.^7^,^14^ Professional billing analysis showed an increase in RVUs per encounter due to the provision of intensive care, but no significant change in overall E/M CPT coding ED billing when excluding ED-ICU encounters and no shift in billing practices. However, there was an increase in net revenue and direct margins per patient encounter post-implementation, suggesting financial benefits for the hospital.^7^

To our knowledge, our investigation and that of Bassin et al are the only two studies to report on the fiscal impact of implementating an ED-ICU. However, direct comparisons between the results of the two are difficult given that the two units operate differently on multiple levels. The unit studied by Bassin et al was foundation funded.^6^,^7^,^14^ In addition, it was separate from the main ED, with intensive care patients initially seen by traditional ED staff before transitioning to the unit. Our study site unit received no external funding and functioned as an integral part of the ED. Patients were either triaged directly to Next Pod for initial assessment or transferred as needed from the main ED. If, and when, patients no longer required critical care-level resources, they were then moved to other areas of the main ED. Thus, there appeared to be greater bidirectional flow than that in the unit studied by Bassin et al. Accordingly, we hypothesize that our observed shift of E/M coding and overall ED billing compared to Bassin et al may have been, in part, a result of improved overall ED operations from the integration of an ED-ICU-type unit within a ED, as opposed to a unit that functioned as a segregated, more independent unit.

With regard to the foundation support for the unit studied by Bassin et al, their unit grew from significant capital investment and increased staffing.^7^ In the case of our study site, the unit grew organically from our generalized, ongoing ED quality and flow improvement initiatives and likely had less financial investment. While there were costs associated with increased staffing (not measured in this investigation), the repurposing of an already functional area within the ED for dedicated high-acuity care required minimal capital investment. This model may represent one more easily adopted by EDs to address the problems of increased acuity and negative effects of increased ICU boarding,^28^–^30^ and could be feasibly implanted by an ED that has a small accessory space/unit with flexible usage.

Operational efficiency can be improved by either increasing RVUs through higher level CPT coding or enhancing patient throughput.^31^ Using CPT codes as a surrogate marker for RVUs per hour, a recognized measure of ED operational effectiveness,^32^–^35^ the data indicated substantial gains in performance, achieved without altering the ED’s physical footprint. By this measure, the unit, while occupying only 4.26% of the total ED square footage, improved overall efficiency.^31^ Additional studies will be required to address other fiscal effects of such units including the cost associated with increased staffing ratios, revenues generated from facility fees, costs associated with potential reduction in overall hospital length of stay, realization of cost-savings associated with changes in overall operational efficiencies of the ED, potential indirect cost and revenue benefits related to medical legal risk changes, and patient experience changes.

## LIMITATIONS

Because the Next Pod was created in a single-site, academic, tertiary-care center; its external reproducibility is unknown.^27^ Since the unit was integrated organically into an already functional area of the ED, accurately isolating the increased staffing costs for meaningful analysis was not feasible. As a retrospective, quasi-experimental study, our investigation did not involve randomization, limiting its ability to clearly demonstrate without possible bias a causal association between an intervention and outcome. Selection bias is also possible via geographic bias as clinicians were treating patients in an ED-ICU setting, although no formal billing education was undertaken. Reporting bias is prevalent in quasi-experimental studies.

This study was subject to the temporal limitations of the COVID-19 pandemic, which substantially affected the study sites ED volume starting in March 2020, limiting timeframe available for data collection that would otherwise have been included. This limitation did not account for the seasonality of ED presentations; however, the control site showed no change in the primary outcome of critical care billing during the study period. It is important to note that the study’s trends continue to be noted after ED volumes have returned to pre-COVID-19 levels and is an active area of ongoing, internal monitoring.

Due to technical electronic health record limitations, patient initial-triage destination was available, but flow in and through the Next Pod could not be differentiated from flow through the entire ED; thus, this study represents the effects on the entire department rather than just those treated in the Next Pod. The flow into, through, and out of an ED-ICU-type unit to optimize efficiency and resource utilization is an integral part of building the operational knowledge base of ED-ICU operations.

## CONCLUSION

The ED-ICU model represents a promising approach to operational and quality improvement in emergency medicine and has been associated with improved patient outcomes. Fiscal sustainability is unknown due to the paucity of literature and questionable generalizability in previous models. In our evaluation of an organically developed ED-ICU, we observed an increased number of critical care charges, higher professional fee revenue, and improved documentation efficiency. Further studies, particularly those examining costs and facility fee revenue, are needed to fully assess the financial performance of an emergency department-based intensive care unit.

## Figures and Tables

**Figure 1 f1-wjem-26-1192:**
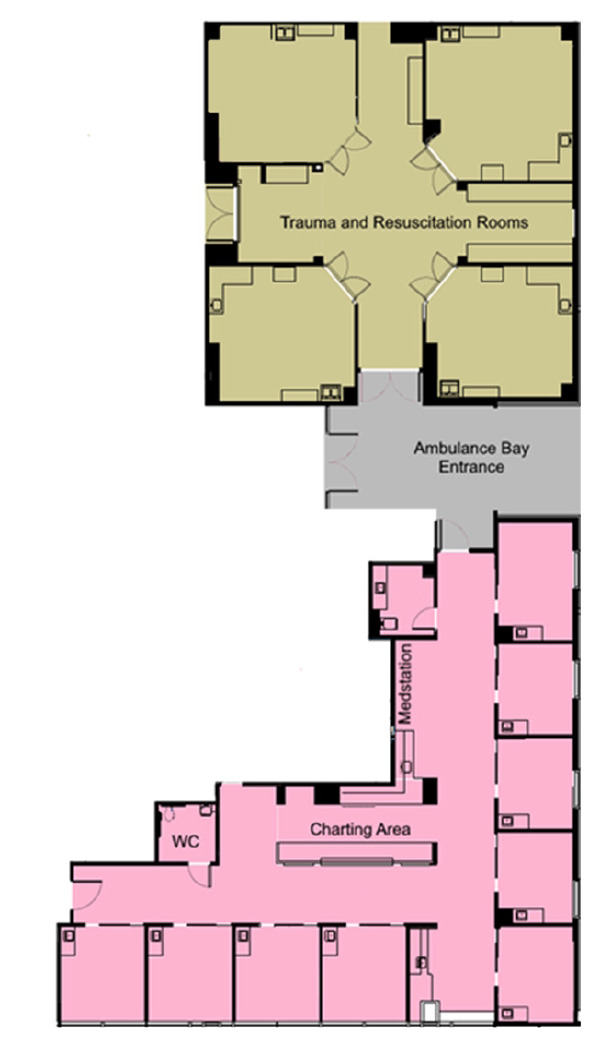
Layout of Next Pod, including resuscitation and trauma rooms. Note that Next Pod area is the ED-ICU. *Next Pod*, North Pod Extension; *WC*, water closet; *ED-ICU, e*mergency department-based intensive care units.

**Figure 2 f2-wjem-26-1192:**
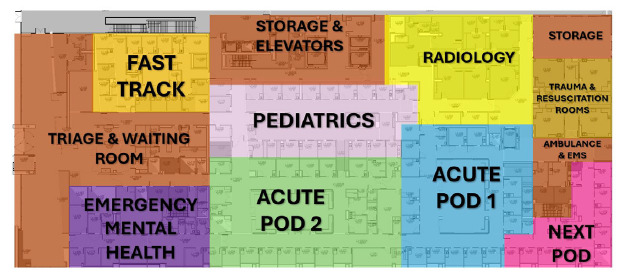
Layout of the study site emergency department. Total ED is 62,264 sq/ft. Next Pod is 2,653 sq/ft (occupying 4.26% of the total ED square footage). Note that Next Pod area is the ED-ICU. *EMS*, emergency medical services; *Next Pod*, North Pod Extension; *ED-ICU, e*mergency department-based intensive care units.

**Figure 3 f3-wjem-26-1192:**
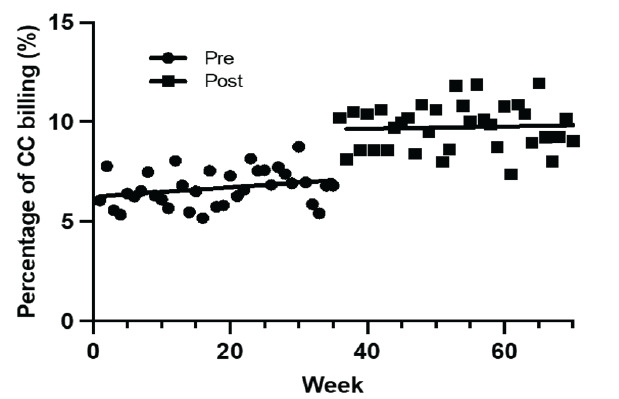
Percentage of ED encounters with critical care time billed at study site before and after the implementation of Next Pod. Note that Next Pod area is the ED-ICU. *CC*, critical care; *ED*, emergency department; *Next Pod*, North Pod Extension; *ED-ICU, e*mergency department-based intensive care units.

**Figure 5 f5-wjem-26-1192:**
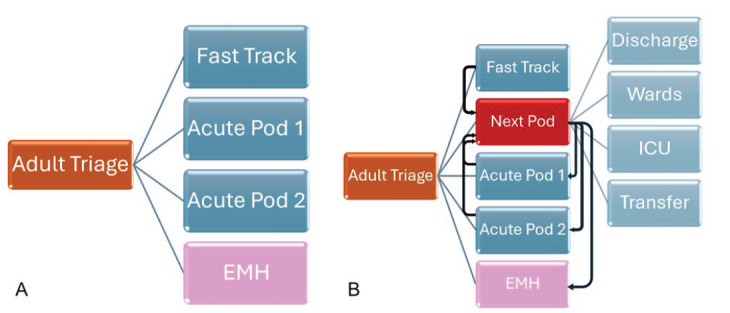
Flow through the emergency department pre- and post-implementation of the Next Pod. Side A shows pre implementation, when trauma/resuscitation rooms were generally covered by Acute Pod 1. Side B shows post implementation where Next Pod generally covered the trauma/resuscitation rooms. Patients were triaged to the Next Pod primarily, or if deemed to need increased critical care resources, were transferred from other parts of the ED. If and when patients were stabilized or downgraded from the perspective of ED critical care resources, patients are then able to be discharged, cleared for EMH, or moved out to the main ED or hospital floors. Note that Next Pod area is the ED-ICU. *EMH*, emergency mental health*; ICU*, intensive care unit*; Next Pod*, North Pod Extension; *ED-ICU, e*mergency department-based intensive care units.

**Table 1 t1-wjem-26-1192:** Patient characteristics.

	Pre-Next Pod (n=38,283)	Post-Next Pod implementation (n=36,424)
Age, mean (SD), y	51.3 (20.1)	51.9 (20.2)
Sex		
Female, %	47.6	47.3
Male	52.4	52.7
Race, %		
Asian	2.2	2.4
Black	7.9	7.8
White	72.4	72.7
Other/Unspecified	17.4	17.1
Ethnicity, %		
Hispanic	16.8	16.4
Not Hispanic	82.2	83.6
Payor, %		
Medicaid	26.5	25.9
Medicare	34.6	34.9
Private	33.2	33.7
Other/Self-pay	5.7	5.5
ESI, mean (SD)	2.85 (0.81)	2.84 (0.82)

* Pre-Next Pod dates: November 11, 2018 – July 14, 2019 (35 weeks).

* Post-Next Pod dates: July 15, 2019 – March 15, 2020 (35 weeks).

Note that Next Pod area is the ED-ICU.

*ESI*, Emergency Severity Index; *Next Pod*, North Pod Extension; *ED-ICU, e*mergency department-based intensive care units.

**Table 2 t2-wjem-26-1192:** Pre- vs post-Next Pod implementation comparison.

Billing data	Pre-Next Pod	Post-Next Pod implementation	*P*-value
Total ED visits, No.	38,283	36,424	-
CPT, No. (%)			
99281	107 (0.3)	89 (0.2)	.35
99282	3,470 (9.1)	2,536 (7.0)	<.001
99283	6,185 (16.2)	4,150 (11.4)	<.001
99284	13,809 (36.1)	13,380 (36.8)	.06
99285	12,160 (31.7)	12,724 (34.9)	<.001
99291	2,372 (6.2)	3,187 (8.8)	<.001
99292*	180 (0.5)	358 (1.0)	<.001
99292 charged >1x, No.†	32	73	.12
Total charges, $	15,556,975	16,255,926	-
Charges, mean, $ (SD)			
All	406 (194)	446 (366)	<.001
CC only	888 (221)	925 (979)	.06
Payment, mean, $ (SD)			
All	140 (96)	152 (102)	<.001
CC only	235 (117)	236 (123)	.79

Pre-ED-ICU dates November 11,2018 – July 14, 2019 (35 weeks).

Post-ED-ICU dates: July 15, 2019 – March 15, 2020 (35 weeks).

* Represents encounters where patients had at least one 99292 charge subsequent to 99291. 99292 charges only counted once, even if charged > 1 time during that encounter.

† Multiple 99292 charges are allowed during a 24-hour period.

Note that Next Pod area is the ED-ICU.

*ED*, emergency department; *CPT*, Current Procedural Terminology; *CC*, critical care; *Next Pod*, North Pod Extension; *ED-ICU, e*mergency department-based intensive care units.
